# *MITF* variants cause nonsyndromic sensorineural hearing loss with autosomal recessive inheritance

**DOI:** 10.1038/s41598-020-69633-4

**Published:** 2020-07-29

**Authors:** Supranee Thongpradit, Natini Jinawath, Asif Javed, Saisuda Noojarern, Arthaporn Khongkraparn, Thipwimol Tim-Aroon, Krisna Lertsukprasert, Bhoom Suktitipat, Laran T. Jensen, Duangrurdee Wattanasirichaigoon

**Affiliations:** 1 Program in Molecular Medicine, Faculty of Science, Mahidol University, Bangkok, Thailand; 2 Research Center, Faculty of Medicine Ramathibodi Hospital, Mahidol University, Bangkok, Thailand; 3 Program in Translational Medicine, Faculty of Medicine Ramathibodi Hospital, Mahidol University, Bangkok, Thailand; 4 Integrative Computational BioScience Center (ICBS), Mahidol University, Salaya, Nakhon Pathom Thailand; 5 Computational and Systems Biology Group, Genome Institute of Singapore, Agency for Science, Technology and Research, Singapore, Singapore; 6 School of Biomedical Sciences, University of Hong Kong, Hong Kong SAR, China; 7 Division of Medical Genetics, Department of Pediatrics, Faculty of Medicine Ramathibodi Hospital, Mahidol University, Rama 6 Road, Bangkok, 10400 Thailand; 8 Department of Communication Sciences and Disorders, Faculty of Medicine Ramathibodi Hospital, Mahidol University, Bangkok, Thailand; 9 Department of Biochemistry, Faculty of Medicine Siriraj Hospital, Mahidol University, Bangkok, Thailand; 10 Department of Biochemistry, Faculty of Science, Mahidol University, Bangkok, Thailand

**Keywords:** Molecular biology, Molecular medicine, Genetics, Genotype, Mutation, Sequencing

## Abstract

*MITF* is a known gene underlying autosomal dominant hearing loss, Waardenburg syndrome (WS). Biallelic *MITF* mutations have been found associated with a rare hearing loss syndrome consisting eye abnormalities and albinism; and a more severe type of WS whose heterozygous parents were affected with classic WS in both cases. The aims of this study were to identify a new candidate gene causing autosomal recessive nonsyndromic hearing loss (ARNSHL) and confirm its causation by finding additional families affected with the candidate gene and supporting evidences from functional analyses. By using whole exome sequencing, we identified a homozygous c.1022G>A: p.Arg341His variant of *MITF*, which co-segregated with the hearing loss in five affected children of a consanguineous hearing couple. Targeted exome sequencing in a cohort of 130 NSHL individuals, using our in-house gene panel revealed a second family with c.1021C>T: p.Arg341Cys *MITF* variant. Functional studies confirmed that the Arg341His and Arg341Cys alleles yielded a normal sized MITF protein, with aberrant cytosolic localization as supported by the molecular model and the reporter assay. In conclusion, we demonstrate *MITF* as a new cause of ARNSHL, with heterozygous individuals free of symptoms. *MITF* should be included in clinical testing for NSHL, though it is rare.

## Introduction

Congenital hearing loss is a prevalent disorder found in 1–2 per 1,000 live births, with more than half of the cases caused by genetic abnormalities^1,2^. Nonsyndromic sensorineural hearing loss (NSHL) accounts for 70% of genetic sensorineural hearing loss (HL), with only 30% being genetic syndromes^1–3^. Common syndromic sensorineural HL (SSHL) includes Usher syndrome, Waardenburg syndrome, and Pendred syndrome^2,3^.

Over 119 nuclear genes are associated with NSHL^2,3^. Seventy six of these genes contribute to autosomal recessive NSHL (ARNSHL), whereas variants in 47 and 5 genes underlie autosomal dominant NSHL (ADNSHL) and X-linked NSHL, respectively^2,3^. Variants in some genes can result in both syndromic and nonsyndromic, and both autosomal dominant and autosomal recessive sensorineural HL. Most families with ARNSHL have severe congenital onset, a negative family history of hearing loss, and consanguinity^1,2^. The most prevalent genetic causes of ARNSHL are *GJB2* and *SLC26A4* variants, accounting for 20% of congenital hearing loss, followed by variants in *MYO15A*, *OTOF*, *CDH23*, and *TMC1*^1–3^. Many variants in mtDNA are also shown to cause maternally-inherited NSHL. Digenic inheritance in NSHL is not prevalent, with the most evident example being mutations in *GJB2* and *GJB6*^4^. Genetic diagnosis of NSHL is a challenging task because of its high genetic heterogeneity.

Next generation sequencing (NGS) is a powerful tool for the identification of new disease-causing gene(s), especially in small pedigrees and sporadic cases. Many studies using targeted exome sequencing have reported various diagnostic rates for hereditary hearing loss based on different populations and approaches with overall diagnostic rate approximately 40%^5–9^. However, given its high heterogeneity, the number of new disease-causing gene variants for NSHL continues to grow.

The human microphthalmia-associated transcription factor (*MITF*) gene is a major regulator of melanocyte development, differentiation, and cell survival^10^. Heterozygous *MITF* mutations cause Waardenburg syndrome, type 2A (WS2A) and Tietz (or Tietz albinism-deafness) syndrome, with autosomal dominant inheritance^10^. WS2A patients exhibit congenital deafness, blue iris, patchy depigmentation of the skin and eyes (white foelock and heterochromia iridis), but no dystopia canthorum^10^. Patients with Tietz syndrome have congenital sensorineural HL, blue iris without heterochromia iridis, and skin and hair color lighter than those of their relatives^10^.

In this study, we aimed to identify a new candidate gene causing ARNSHL and confirm its causation by finding additional families associated with this gene using our in-house targeted exome sequencing in a cohort of 130 individuals affected with NSHL. We also performed multiple functional analyses to find supporting evidences. We demonstrate *MITF* as the cause of ARNSHL, with heterozygous individuals free of symptoms, as supported by findings of two families and evidences from functional characterization. Our data also suggest that *MITF* is a rare cause of ARNSHL.

## Results

### Clinical phenotypes of Family-1 and clinical exome data

**Figure 1:**
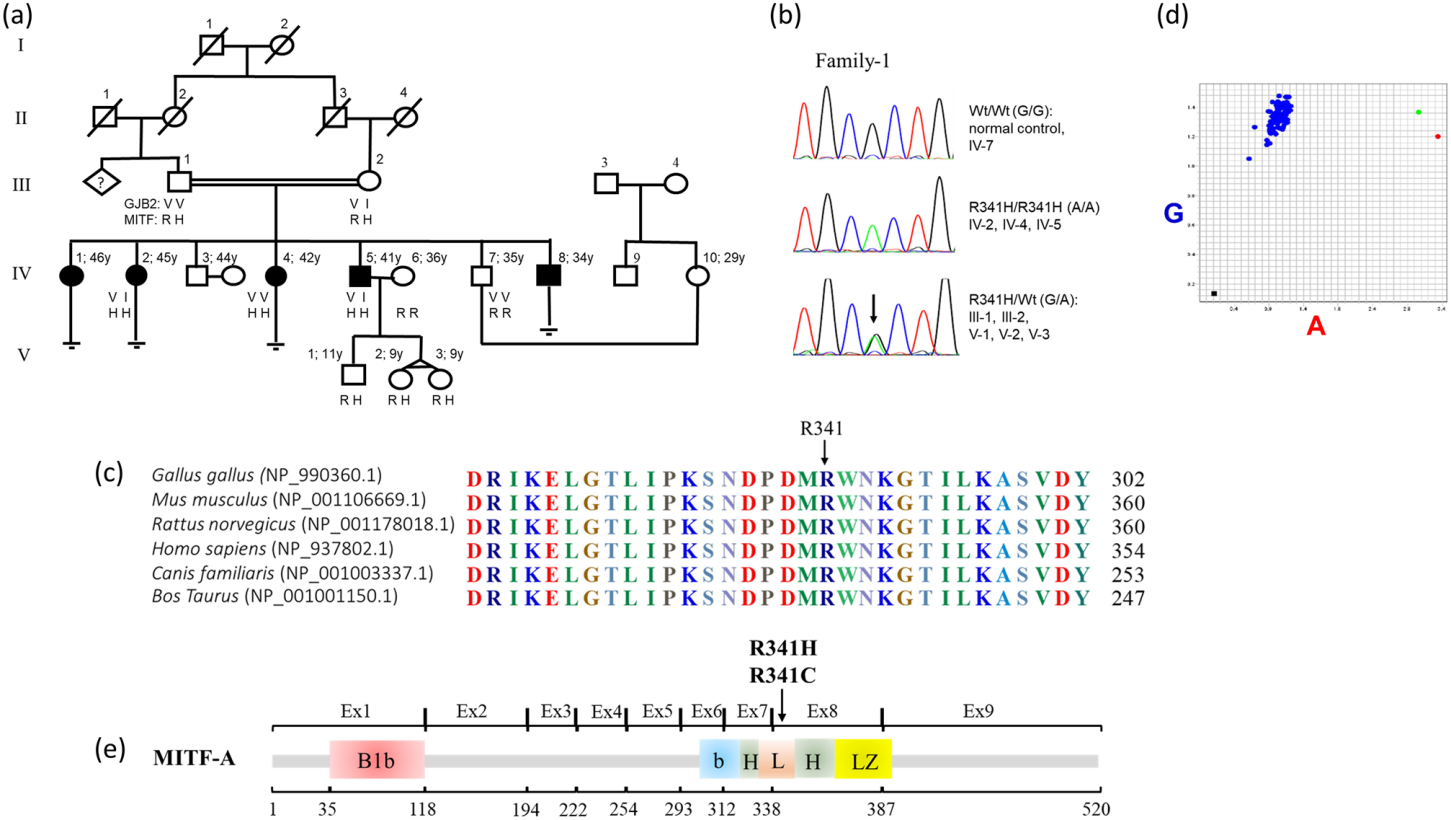
Family-1 and Arg341His. (**a**) Pedigree showing consanguinity between individual III-1 and III-2. Black-filled squares and circles indicate hearing impaired males and females, respectively. (**b**) Chromatogram displaying wild-type (GG), homozygous (AA), and heterozygous (GA) at nt 1,022, with the homozygous variants co-segregating with hearing loss phenotype. (**c**) Protein sequence alignment of vertebrate *MITF*; noted the highly conserved Arg341 residue across various species. (**d**) Allelic discrimination plot obtained from real-time PCR using TaqMan SNP genotyping of *MITF* c.1022G/A. The allelogram showing the blue, green, and red dots representing specimens with GG, GA, and AA genotypes, in orderly; and the black square (left lower corner) denoting a blank control. The GA- and AA-positive specimens were from carrier and affected individuals of Family-1, respectively. Among 228 control individuals screened, only the wild-type GG genotype was found. (**e**) Arg341His/Cys is located in the loop segment of the basic helix-loop-helix (bHLH) domain of MITF-A.

Family-1 consisted of a consanguineous hearing couple, five deaf and two normal hearing children. Affected individuals IV-1, IV-2, IV-4 and IV-5 were found to have deaf-mutism (Fig. 1a). They were normal on physical examination of color of hair, iris and skin; inner canthal distances; thyroid glands; visual acuity as screened by the Snellen chart; and visual field confrontation using a simple Donders’ test. There was no known history of recurrent syncope, thyroid, cardiac, or kidney problems. None had auditory rehabilitation. Complete blood counts, thyroid function tests, blood urea nitrogen and creatinine levels, and urinalysis all showed normal results. Individual IV-1 refused to have blood taken.

Dilated fundal examination, an audiological study, and a temporal bone CT scan were declined by the affected individuals. Both parents were normal on physical examination and blood and urine tests were normal. They reported no substantial health problem or premature greying of hair. Based on this data, we concluded that this family represents profound congenital NSHL inherited in an autosomal recessive manner.

Targeted exome analysis using a commercially available gene panel (70 genes; OtoGenome) revealed heterozygous p.Val37Ile *GJB2* mutation in the affected individual IV-5. This analysis confirmed our previous finding and supported the exclusion of *GJB2* and the other genes in the panel as the cause of NSHL in this family. We therefore aimed to identify a new candidate gene.

### Whole exome sequencing (WES) data of Family-1

Specimens from affected individuals (IV-2, IV-4 and IV-5) and an unaffected sib (IV-7) were chosen for WES. We first filtered for homozygous variants in each patient and for heterozygous variants in the unaffected sib. Variants on chromosomes X and Y; synonymous SNVs; likely non-disease-causing variants (intronic, intergenic, 3′-UTR, 5′-UTR, downstream, upstream and in ncRNA), and common variants present in the 1000Genome database with allele frequency > 0.03 were discarded (Table 1). These steps left 36, 43, and 31 homozygous insertion, deletion, and exonic and splicing variants in affected individuals IV-2, IV-4, and IV-5, respectively. Among these, only 28 variants were shared by all three affected sibs. Twenty-seven out of 28 genes were also present in the homozygous state in the unaffected sib, excluding them as being the causative gene. The single variant that remained and that was absent in the unaffected sib was a novel *MITF* allele, c.1022G>A: p.Arg341His in exon 9 of MITF-isoform A (NM_198159.2), corresponding to c.719G>A: p.Arg240His in exon 8 of MITF-isoform M (NM_000248.1).

**Table 1:** Number of variants remaining after stepwise filtering.

Filtering criteria	Number of variants remaining			
	IV-2	IV-4	IV-5	IV-7
Total variants	73,535	71,858	72,274	72,620
Homozygous variants only	35,173	34,222	35,563	NA
Heterozygous variants only	NA	NA	NA	37,401
After excluding variants on chromosome X and Y	34,424	33,481	34,617	37,318
After excluding synonymous SNVs	29,963	28,992	30,053	31,816
Insertion/deletion, exonic, splicing onlya	4,277	4,195	4,397	5,316
After excluding variants in 1000Genome with AF > 0.03^b^	36	43	31	119
Homozygous variants shared among affected persons	28^c^	28	28	NA
Homozygous variants shared among affected persons, but heterozygous/wild-typed in the hearing subject	1	1	1	NA

*AF* allele frequency, *NA* not applicable.

^a^Excluding intronic, intergenic, 3′-UTR, 5′-UTR, downstream, upstream and ncRNA.

^b^As of Oct 2011.

^c^27 genes shared by all three affected but not shared with the unaffected sib include *ATXN1*, *CDCP2, FOXI3, KIF1A, CCDC66, DCP1A, EOMES, SEMA3B, FBXL21, SRA1, MAP3K4, TBP, MAFA, PRUNE2, EFEMP2, MAML2, TMEM132A, PLBD1, IFI27, RIN3, OTUD7A, PCSK6, BPTF, COPZ2, KCNC3, RALY, PRDM15.*

Phen-Gen analysis indicated *MITF* as the most probable candidate gene as it had the highest damaging score of 0.0885690996, while the genes ranking 2–17 had far lower damaging scores, ranging from 0.0055963440 to 0.0000022778 (Supplementary Table S2).

Moreover, the analysis of 144 known hearing loss genes in each individual WES data using compound heterozygosity model was also carried out. The analyses led to an identification of 16 variants in 15 genes. Among these variants, only three were pathogenic or likely pathogenic according to the 2015 American College of Medical Genetics (ACMG) guideline for variant classification^11^. These included heterozygous p.Val37Ile of *GJB2* in two affected individuals; heterozygous p.Pro1791Arg of *TECTA* in one deaf individual; and p.Arg341His of *MITF* gene in all three affected persons. Compound heterozygosity or homozygosity of pathogenic/likely pathogenic mutations other than of the *MITF* variants was not found (Supplementary Table S1). In addition, we checked the exome data for potential missing call/segment, by using IGV and did not identify lost alleles (Supplementary Figure S1).

### Homozygous Arg341His co-segregated with hearing loss phenotype in Family-1

Sequencing of additional family-1 members confirmed the affected individuals to be homozygous for *MITF* Arg341His, both parents to be heterozygous for *MITF* Arg341His, and hearing relatives to be wild-type or heterozygous for *MITF* Arg341His (Fig. 1b). These data further support the hypothesis of autosomal recessive inheritance of HL in this family. Bioinformatic analysis indicated a likely pathogenic property of p.Arg341His, with a SIFT score of 0.00, a PolyPhen score of 1.00, a MutationTaster score of 0.99^+^, and no splicing defect (splice score 0.99).

Alignment of multiple vertebrate protein sequences (chicken, mouse, rat, human, dog, and cow) using Clustal Omega Software (https://www.ebi.ac.uk/Tools/msa/clustalo) revealed high conservation of residue 341Arg (Fig. 1c). p.Arg341His was not present in 756 control chromosomes of the in-house WES database, as screened by TaqMan assays (Fig. 1d). This variant is located in the loop segment (aa 332–346 isoform MITF-A) of the basic helix-loop-helix (bHLH) domain of MITF (Fig. 1e)^12^.

### Additional family and Arg341Cys *MITF* mutation

**Figure 2:**
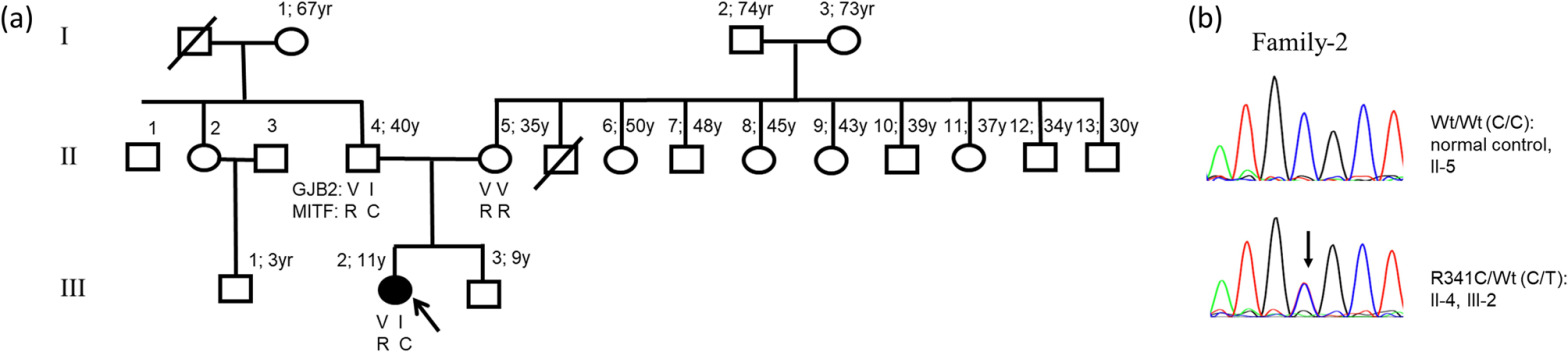
Family-2 and Arg341Cys. (**a**) Pedigree, black-filled circle indicates hearing impaired female, respectively. (**b**) Sanger sequences showing wild-type and heterozygous C to T at nt1021; and the heterozygous variant found in the patient and father only.

By screening 130 individuals affected with NSHL using our in-house targeted exome sequencing, it revealed another family (Family-2) with a sporadic patient who had a heterozygous *MITF* mutation, c.1021C>T: p.Arg341Cys of isoform MITF-A, and a heterozygous *GJB2* mutation, c.109G>A: p.Val37Ile (Fig. 2). The patient (individual III-3), an 11-year-old girl, had normal colored iris and fine, sparse and reddish-brown hair. The remaining physical examinations were normal. The patient was noted to have profound (110 dB) sensorineural hearing loss at age 9 months and received a cochlear implant at the age of 3 years. Blood and urine tests indicated normal kidney and thyroid functions. A temporal bone CT scan was normal. Both parents appeared normal and reported no pigmentation abnormality in either side of the family. WS and Teitz syndrome were unlikely because there was no blue iris or patchy depigmentation in either the patient or the father. In addition, fine and sparse hair is not a feature of either syndrome. We, therefore, concluded that this patient had NSHL.

The in silico analysis indicated likely pathogenicity of p.Arg341Cys with no effect on splicing (SIFT score 0.00; PolyPhen score 1.00; MutationTaster score 0.99^+^; and splice score 0.99). This variant was not present in our control WES database.

To identify other *MITF* or *GJB2* mutations possibly missed by the gene panel testing, we performed PCR-Sanger sequencing of all ten *MITF* and two *GJB2* exons in the patient and both parents (Supplementary Tables S3 and S4). The results confirmed that the patient and her father were double heterozygous for *MITF*-Arg341His and *GJB2-*Val37Ile, while the mother carried no mutation. Moreover, the copy number variation (CNV) analysis performed in the affected individual of Family-2 did not reveal microdeletion/duplication of chromosomal segment involving the *MITF* gene. Additionally, PCR-sequencing of the *TYR* gene was performed and revealed no pathogenic variant (Supplementary Table S5).

### Functional consequence of the Arg341His and Arg341Cys *MITF* mutations

After *MITF* was revealed as a new candidate, we performed reporter, Western blot, and immunofluorescence assays to investigate the functional consequences of the identified variants, using melanoma (G361) and mouse embryonic fibroblast (NIH/3T3) cells.

**Figure 3:**
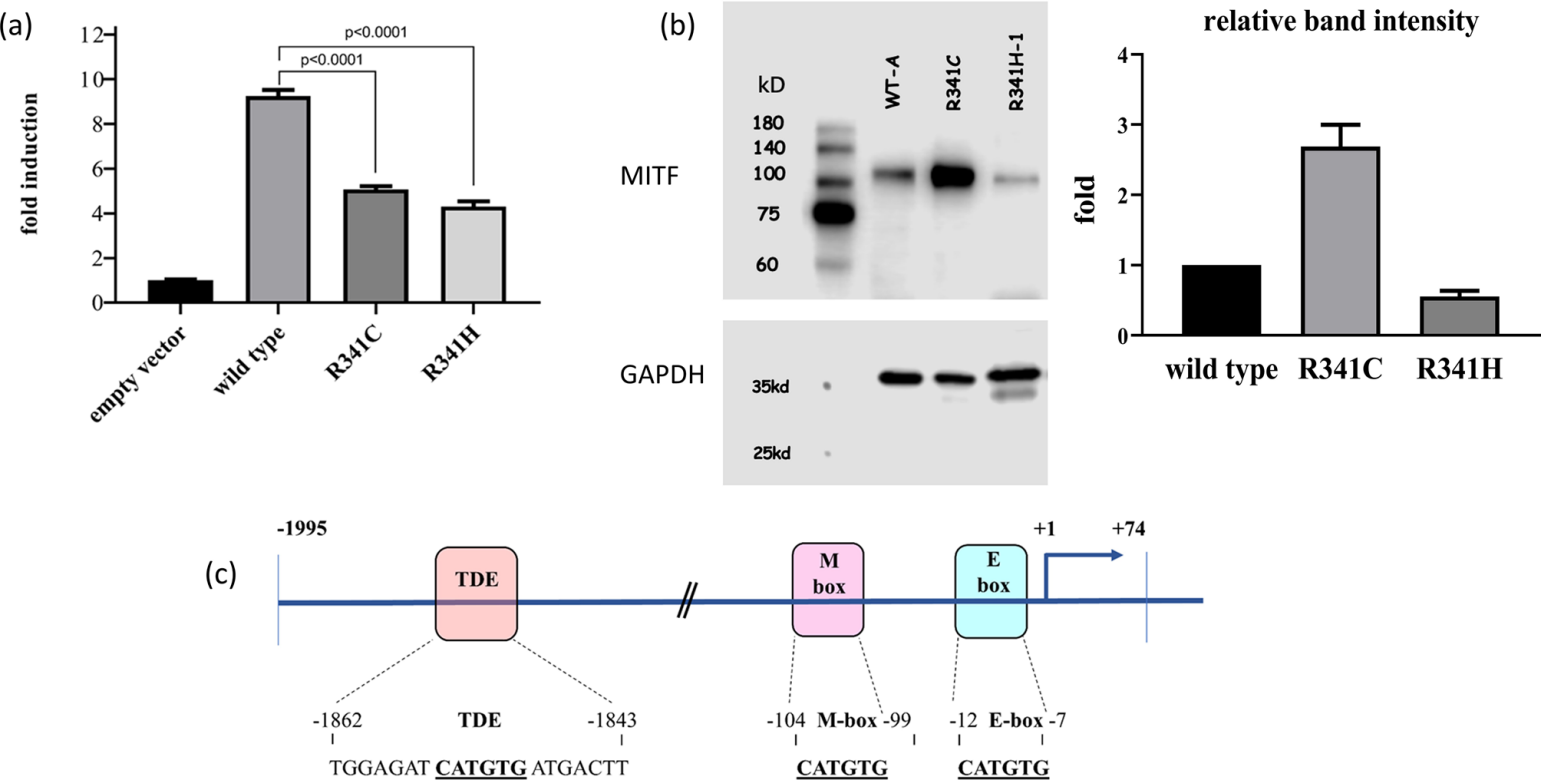
Reporter assay and western blot analysis (**a**) The basal luciferase level of the empty vector was 32. Overexpression of wild-type MITF induced *TYR* promoter activity by up to 9.4-fold (300/32), R341C at 5.1-fold (164/32 or 54%), and R341H at 4.3-fold (139/32 or 46%), respectively, (p < 0.0001 by one-way ANOVA)*.* (**b**) Western blot analysis. Left panel: upper image demonstrating the blot of MITF, from left to right, molecular size marker; wildtype MITF-isoform A, R341C, and R341H variants (labeled as R341H-1), the wild-type, R341C and R341H protein with normal predicted size. Lower image of the left panel showing the blot of GAPDH used as a loading control for normalization for wildtype MITF and each variant. Noted almost equivalent amount of the GAPDH in all lanes. Detail of GAPDH staining was described in the Supplementary Figure S1. Brightness and contrast of the upper and lower images were not modified from its original appearances The right panel denoting a greater amount of the R341C protein and a decreased quantity of the R341H protein expressed, compared with the wild type [*p*-value 0.0011 and 0.0898, respectively, using ANOVA with post hoc (Tukey) test]. The relative level of band intensity of wild-type MITF was set as 1. (**c**) Diagram showing the Luc-reporter constructs, with putative MITF binding sites shown as a filled box (Tyrosinase distal enhancer or TDE, M-box, E-box).

**Figure 4:**
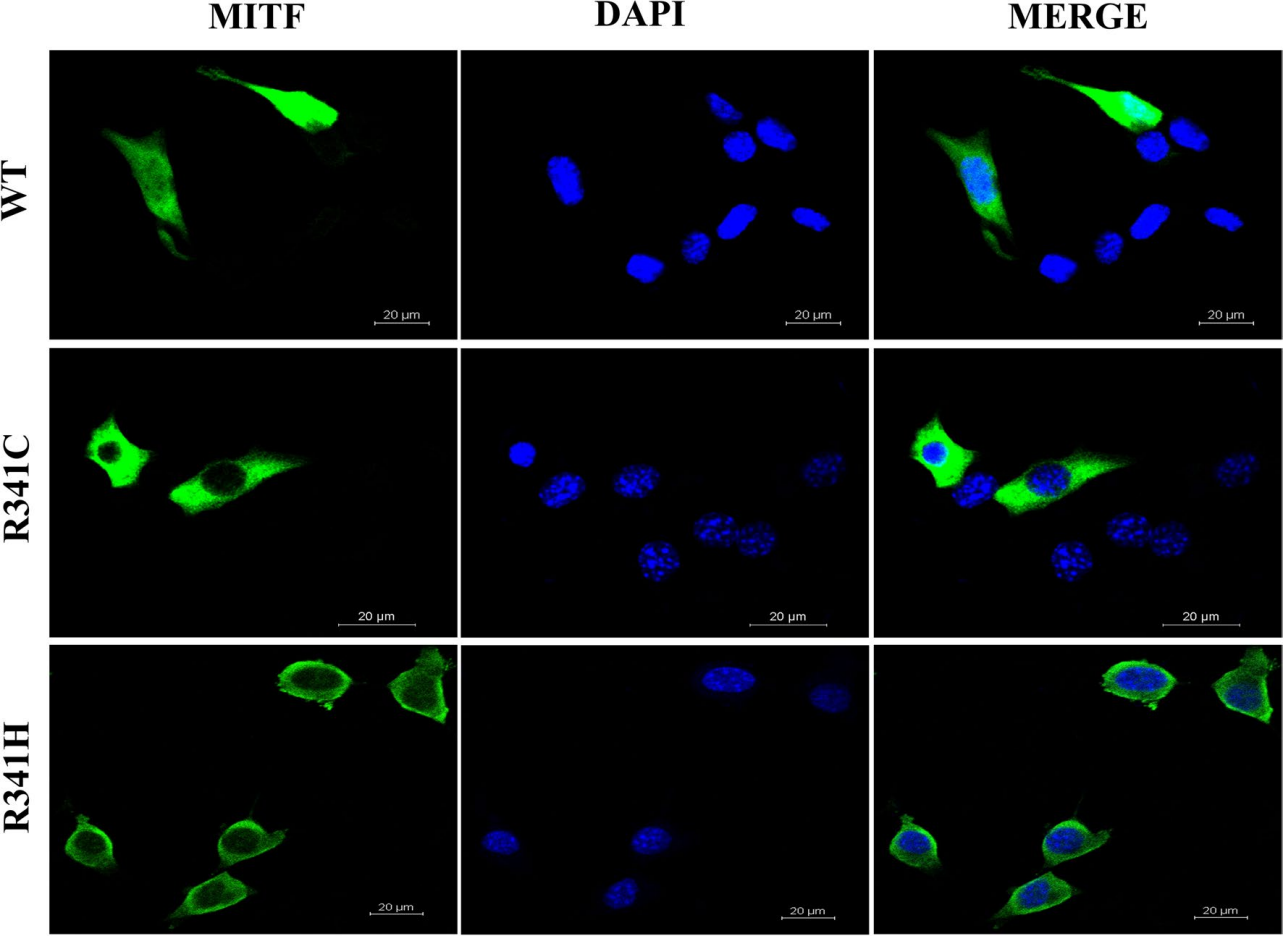
Subcellular localization of wild-type and mutant MITF proteins. The studied proteins are in green (left panel), DAPI-stained nuclei are shown in blue (middle panel), and the merged images are shown on the right panel. Note the nuclear localization of the wild-type MITF and the exclusively cytosolic localization of the R341C (Arg341Cys) and R341H (Arg341His) variants.

Overexpression of Arg341His and Arg341Cys induced *TYR* promotor activity to only 46% and 54% of the wild-type expression level, respectively (Fig. 3a). Western blotting revealed normal sized Arg341His and Arg341Cys MITF proteins but a significantly increased amount of the Arg341Cys protein was expressed and a slightly decreased quantity of the Arg341His variant was expressed compared with wild-type MITF (Fig. 3b and Supplementary Figure S2). An immunofluorescence study indicated that both Arg341His and Arg341Cys MITF were localized solely in the cytoplasm compared with nuclear localization of wild-type MITF (Fig. 4).

### Molecular model of Arg341His and Arg341Cys

**Figure 5:**
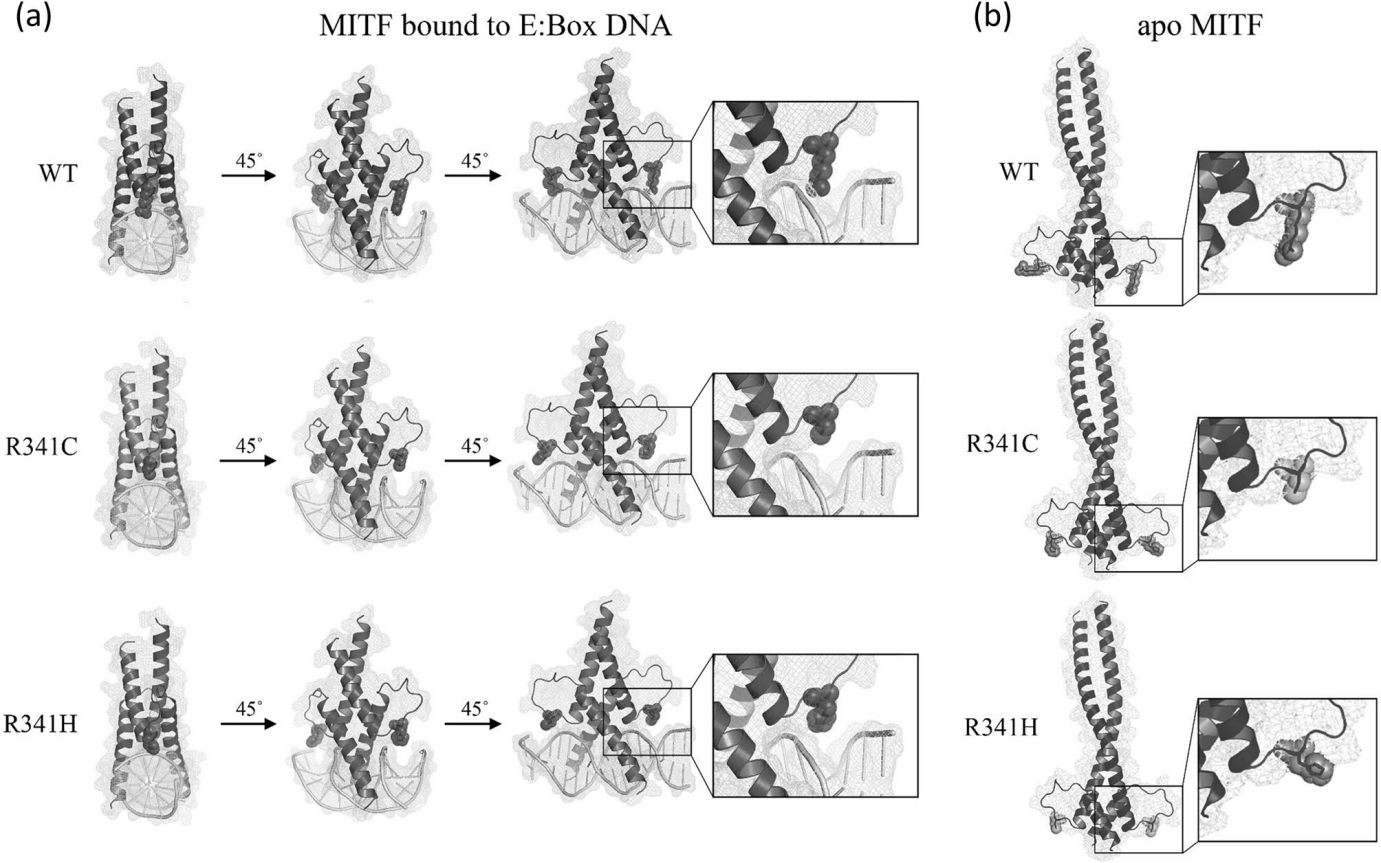
Molecular modelling of wild-type, Arg341Cys, and Arg341His MITF. Homology models of wild-type, Arg341Cys, and Arg341His MITF constructed using *Mus musculus* MITF:E-box complex (4atk) and apo MITF (4ath) as templates^.^ The Arg341 residues, as well as Cys and His substitutions, are shown with space-filled visualizations. (**a**) The model of MITF bound to DNA contains residues 308–369. The three views are 45° rotations of the model. The boxed region is magnified to show DNA interaction details of Arg341 and the loss of these contacts in the Arg341Cys and Arg341His mutants. (**b**) The model of apo MITF contains residues 318–396. One view is shown with the region surrounding residue 341 magnified. SWISS-MODEL was used to generate the structural models of wild-type and mutant MITF proteins which then were viewed using the PyMOL Molecular Graphics System, version 2.0.6 (https://pymol.org/2/).

The potential effect of substitutions at Arg341 was examined using homology models for MITF bound to the E-Box DNA element as well as the apo form. This analysis indicated that Arg341 (MITF-A isoform) is not present at the dimer interface in either the apo or DNA bound forms (Fig. 5). Instead, Arg341 is located near the DNA binding domain and makes contacts with the E-Box DNA element. The corresponding residue in MITF-M, Arg240, has been reported to not make base specific contacts but interacts with the phosphate backbone on the E-Box element^10^. The Arg341Cys and Arg341His substitutions abrogate this contact between MITF and the DNA. Thus, mutation of Arg341 may reduce the binding affinity of MITF to this DNA element, although the impact of loss of the Arg341 contact with the sugar-phosphate backbone is unknown.

## Discussion

We demonstrated *MITF* to be a new causative gene for ARNSHL based on the following evidence: (1) other known genes were excluded; (2) *MITF* variants were a recurrent phenomenon in multiple affected families; (3) cosegregation of the homozygous variants with hearing loss, in the absence of homozygous mutations in hearing relatives and a control population; (4) deleterious effects of the mutations, as shown by in silico analysis and molecular modeling, disrupted expression of a downstream gene, and aberrant subcellular localization; and (5) the known roles of *MITF* in cochlear melanocytes. Two *MITF* mutations were identified: p.Arg341His and p.Arg341Cys.

Only four individuals from Family-1 were selected for WES. The following selection strategies were considered: (A) two affected sibs and both parents; (B) two affected sibs and one unaffected sib and one parent; and (C) three affected sibs and one unaffected sib. For option A, the chance of two affected sibs sharing any homozygous variant while both parents were heterozygous was 6.25% or 1/16 (1/4 × 1/4 × 1 × 1), regardless of the variants being benign or pathogenic. For option B, the chance of two affected sibs sharing a homozygous variant whilst the parent being a carrier and the unaffected sib not harboring the homozygous variant was 4.69% or 3/64 (1/4 × 1/4 × 1 × 3/4). For option C, the probability of three affected sibs sharing a homozygous variant while the unaffected sib carrying a heterozygous or wild-type sequence was 1.17% or 3/256 (1/4 × 1/4 × 1/4 × 3/4). Therefore, we chose option C, which provided the lowest chance of a false candidate.

In Family-1, besides *MITF*, none of the genes detected by Phen-Gen overlapped with those filtered by manual analysis. By using Phen-Gen, we can avoid human error when filtering the data of each individual before segregation analysis. Phen-Gen has a 52% advantage over a genotype-only approach^13^.

Hearing impaired individuals in family-2 is likely having recessive mutations on both alleles of the *MITF* gene, p.Arg341Cys inherited from the father and another yet unidentified pathogenic allele which could be inherited from the mother or a de novo variant. Based on this assumption and the result of DNA binding activity of the p.R341C, it supports that family-2 also fits with the autosomal recessive model.

Human MITF binds DNA as a homodimer and as a heterodimer with the TEE family of bHLHZip transcription factors^10^. Over 77 *MITF* mutations have been reported to cause WS2A and Teitz syndrome (HGMD https://protal.biobase) with around 50% being missense variants. Truncating and splice site *MITF* mutations occur throughout the gene while missense and in-frame mutations were more confined to exons 8 and 9 of MITF-A isoform (or exons 7 and 8 for MITF-M)^14^. Functional analysis data of *MITF* variants are limited. A recent study showed that missense (p.Arg217Ile) mutations of *MITF* resulted in WS2A through a dominant negative effect, whereas a frameshift mutation c.575delC (p.Thr192LysfsTer20) showed a haploinsufficiency mechanism^15^.

The significant reduction of downstream *TYR* gene expression caused by Arg341His and Arg341Cys supported the pathogenic nature of these *MITF* variants (Fig. 3a). The normal molecular weight of the Arg341His/Cys MITF variants confirmed no protein truncation associated with these changes. We could not explain why and how the Arg341Cys led to increased protein expression of the mutant allele.

The Arg341 residue is present on the exterior of the MITF protein in both apo and DNA bound forms. This suggests that Arg341Cys and Arg341His mutations are unlikely to have a significant impact on the overall structure of MITF. In addition, Arg341 is not present within the dimer interface indicating that dimer formation is unlikely to be altered due to the Arg341Cys and Arg341His mutations. While Arg341 does make contacts with the sugar-phosphate backbone of DNA when bound to the E-Box element it is unlikely that loss of this interaction alone promotes cytoplasmic localization of MITF. No association between loss of DNA binding and cytoplasmic localization has been demonstrated. Mutant forms of MITF lacking the complete DNA binding domain are retained within the nucleus^16^. Instead it seems possible that Arg341 may be part of a nuclear localization signal. Basic regions surrounding Arg341 (corresponding to Arg240 in MITF-M) have been demonstrated to promote nuclear localization. Residues in MITF-M that enhance nuclear localization include Arg and Lys residues between amino acids 197–206 and 255–265^15–17^. Mutations of Arg and Lys residues within either of these regions increase the fraction of MITF in the cytosol, similar to the effect seen with Arg341Cys and Arg341His variants. The identification of Arg341His was novel when we discovered it but it has since been listed as a variant of unknown significance for a non-specified condition in the ClinVar database (VCV000505101.1).

The *MITF* gene functions to regulate melanin production through tyrosinase and is also directly involved in the molecular process of hearing. In the inner ear, melanocytes are present in the intermediate layer of the stria vascularis. These melanocytes, so called intermediate cells possess a functional Na^+^-K^+^-ATPase, which controls the ion and material transport necessary for the maintenance of normal volumes and ion concentrations (recycling) of the endolymph. Therefore, it is essential in regulating the integrity of the stria vascularis and for maintaining normal hearing thresholds^18^. Only melanocytes in the inner ear possess this function whereas melanocytes in other pigmented tissues lack such a role^18^. In mice, depletion of the intermediate cells causes a substantial reduction in endocochlear potentials, severe degeneration of the endolymphatic space, and collapse and loss of sensory hair cells, accompanied by significant hearing loss^18,19^.

Mutations of *MITF* yielding diverging mechanisms but resulting in a similar phenotype have been recently demonstrated in melanoma, in that MITF knockdown and overexpression in murine and human melanoma cells showed opposing mechanisms, decrease or increase of immune cell migration and vice versa^20^. In our case of HL, different mechanisms of *MITF* mutations, impaired Na^+^-K^+^-ATPase function only or with defective melanin production, could lead to the same phenotype of hearing defect, though with some phenotypic variation, nonsyndromeic or syndromic type. This could be attributed to differences in genetic background, potential genetic redundancy or compensation of other associated genes depending on the defective mechanism involved of the particular *MITF* variant(s), and diverging individual mutations (dominant negative effect, haploinsufficiency or loss-of-function) rather than the gene itself.

Due to loss of function and Arg341His/Cys being located outside the DNA binding domain, it is unlikely that heterozygosity for Arg341His/Cys would disrupt DNA-binding ability. It is, therefore, very unlikely to disturb the neural crest-derived cochlear melanocytes in the early embryo stage or to cause the WS2A phenotype^10^.

Homozygosity for Arg341His/Cys may not disrupt in vivo* TYR* expression to below the critical level that can lead to albinism phenotypes. In addition to *MITF*, there are other genes, such as *OTX2*, *TP53*, and *LEF1*, that regulate *TYR* gene expression^21–23^. It is possible that the deleterious effect of p.Arg341His/Cys *MITF* on *TYR* expression can be overcome by the aforementioned genes. In contrast, a lack of genetic compensation for Na^+^-K^+^-ATPase function in cochlear monocytes can disrupt the endocochlear potential (EP) and results in NSHL^24,25^.

To our knowledge, this is the first time that biallelic mutations of *MITF* have been shown to cause NSHL with autosomal recessive inheritance, with heterozygous individuals free of symptoms. Biallelic *MITF* mutations were also shown to cause the rare syndrome, COMMAD (coloboma, osteopetrosis, microphthalmia, macrocephaly, albinism—oculocutaneous type, and deafness) in two unrelated children whose heterozygous parents were affected with classic WS2A^26^^,^ while children in another family had a WS4-like phenotype but with more severe depigmentation of hair and skin while their parents were mildly affected^27^. Incomplete dominance was proposed as a mode of genetic inheritance for these two families^26,27^.

There are examples of severe phenotypes caused by heterozygous mutations, through gain of function or dominant negative mechanisms, whereas homozygous variants of the same gene can lead to a less severe phenotype due to loss of function. One example associated with HL is heterozygous *GJB2* (*Cx26*) mutations resulting in an autosomal dominant syndromic HL, keratitis-ichthyosis-deafness (KID), and a homozygous *GJB2* mutation causing ARNSHL^28–30^.

There are a number of limitations to the present study. Firstly, a thorough ophthalmological examination and temporal bone CT scan was not performed on affected individuals from Family-1; therefore, concealed associated defects cannot be completely ruled out. Secondly, cellular physiology was not studied to investigate the effect of the mutant MITF on Na^+^-K^+^-ATPase functions. Thirdly, the unidentified allele in Family-2 could be missed because WES/gene panel study and conventional sequencing cannot detect point mutation in the promotor region or deep introns, and the platform of CNV used in this study is not designed with dense probe for detection of a single/few exons deletion/duplication.

NSHL due to digenic inheritance between *MITF* and *GJB2* genes was previously proposed in a single sporadic patient with NSHL^31^. Our data does not support digenic inheritance between *MITF* and *GJB2*, at least for Arg341His/Cys paired with Val37Ile because the HL phenotype was not observed in several individuals with this double heterozygosity. The presence of the *GJB2*-Val37Ile allele in our patients could represent a coincidental finding because the Val37Ile variant is prevalent in the Thai population with an allele frequency of 8.5%^32^. A different mutation at the codon 341, heterozygous p.Arg341Gly compounded with an unidentified mutation in the other allele has been reported in two hearing-impaired siblings from a consanguineous family, whereas both parents showed normal physical and hearing phenotypes, and only the mother carried Arg341Gly^33^.

In conclusion, we demonstrated that *MITF* can cause ARNSHL. Our data expand the list of genes underlying NSHL and indicate a new inheritance pattern of *MITF*. Digenic inheritance between *MITF* and another gene is possible and requires further characterization.

## Methods

### Patients and clinical exome analysis

Affected individual, IV-5, was previously evaluated and found to be heterozygous for a p.Val37Ile (c.109G>A) *GJB2* allele but to not carry the *GJB2* IVS1 + 1G>A variant. Mutation analysis including gap PCR for the 342-kb deletion del*GJB6*-D13S1850 and del*GJB6*-D13S1854 of *GJB6* (*Cx30*), and direct sequencing of *GJB3* (*Cx31*), *SLC26A4*, and *MTRNR1* (a 12s rRNA gene: mtA1555G and mtC1494T) revealed no pathogenic variants^34,35^. DNA from individual IV-5 was analyzed using a hearing loss gene panel consisting of 70 HL genes (OtoGenome, Laboratory for Molecular Medicine, Cambridge, MA, USA, Supplementary information). This analysis identified the heterozygous p.Val37Ile mutation, validating our initial finding and supporting the exclusion of *GJB2* and the other genes in the panel as underlying HL in this family. We therefore aimed to identify a new candidate gene.

Following identification of the candidate gene, we conducted a separate parallel mutation screening in a cohort of 130 individuals affected with early onset NSHL, using our in-house gene panel comprising 64 genes, including the candidate gene.

Each participating subject provided written informed consent, following approval of the protocol by the Ramathibodi Hospital Human Research Ethics Committee (protocols ID 07-56-66; 11-57-29; and 1324; MURA2020/367). All the experiment protocols involving humans were in accordance to international guidelines of Declaration of Helsinki.

### Whole exome sequencing and analysis

DNA were subjected to WES using Illumina HiSeq 2000 (Macrogen, South Korea) with Agilent’s SureSelect for target enrichment and 100 bp Pair End mode (125× coverage). The exome data were quality assessed with the FastQC package and by read alignment against a reference genome (hg19 from UCSC genome browser database) using BWA version 0.5.9 (SAMTOOLS for variant identification; ANNOVAR for variant annotation).

Variants having minor allele frequencies > 0.03 in the 1,000 Genomes Project (November 2010 and October 2011 releases) and in our in-house database of 150 Thai exomes were filtered out. Only rare functional variants, including missense, nonsense, frameshift, non-frameshift, and splice site variants, were kept then separated into two groups, homozygous and heterozygous alleles, for subsequent manual inspection. Two scenarios were applied as follow: first, homozygous mutation in affected individuals, and heterozygous or wild-typed for unaffected persons; and second, compound heterozygous mutations for affected offspring, and heterozygous or wild-typed for unaffected persons. A total of 144 known hearing loss genes were analyzed (Supplementary information).

Computational (in silico) predictive programs, namely SIFT, PolyPhen-2, MutationTaster, Splice Site Prediction by Neural Network; and mutation/variant databases including ClinVar and the Human Genome Mutation Database (HGMD), were used to determine pathogenicity of the variants identified. Sanger sequencing was performed to verify the variants detected.

Phen-Gen online tool was used to identify causative genes. Individuals’ annotated variant files were combined into one file using CombineVariants tools (GATK, Genome analysis toolkits) and the phenotype data file (PED) was created using PLINK application^13^. The standard human phenotype ontology, congenital sensorineural hearing impairment with HPO:0008527 was employed. The new combined genotype file, HPO and PED files were then submitted together to PHEN-GEN (https://phen-gen.org/).

### Screening for mutation(s) identified in a control population

A real-time PCR (Taqman) assay was performed on 228 DNA specimens from regular blood donors, using 7,500 Real-Time PCR System (Applied Biosystems). The hydrolysis probes and primers were designed according to the *MITF* gene sequence (GenBank NM_198159.2). The probes for detecting the wild-type and the Arg341His mutant alleles were tagged with 6-FAM and VIC, respectively, at the 5′ end. The primer and probe sequences were as follow: Forward primer 5′-GTTTTCCTCCATTTTCATCGCAGAG-3′; Reverse primer: 5′-TCCACGGATGCTTTAAGATG-GTT-3′; wild-type probe: 6-FAM 5′-CTTGTTCCAGCGCATGT-3′ MGB; mutant probe: VIC 5′-CCTTGTTCCAGTGCATGT-3′ MGB.

Each real-time PCR reaction contained 20 ng of gDNA, 0.9 μM of each primer, 0.25 μM of each probe and 5 μl of TaqMan Genotyping Master Mix (Applied Biosystems). The thermal profile included initial denaturation at 95 °C for 10 min, followed by 40 amplification cycles of denaturation at 92 °C for 15 s, and annealing and extension at 60 °C for 60 s.

Our in-house WES database from 150 unrelated individuals affected with disorders other than HL was checked for the presence of the variants identified. Reference sequences were as follow: NT_011520.11, NM_006941.3, NP_008872.1 for MITF isoform A; and NT_022495, NM_000248, NP_000239 for MITF isoform M.

### Copy number variation (CNV) analysis

CNV analysis using single nucleotide polymorphism array (Illumina Infinium CytoSNP-850K BeadChip) was performed then analyzed by using BlueFuse Multi software v4.1.

### Targeted-exome sequencing using our in-house gene panel

DNA was extracted from patients’ peripheral blood using Purgene DNA extraction kit. A 20 ng of genomic DNA were subjected to NGS using Ion AmpliSeq Designer (Thermo Fisher Scientific) with 100% coverage for all coding sequences of 64 HL associated genes. The targeted sequencing was performed using Ion Torrent system (Thermo Fisher Scientific) as per manufacturer's instructions. The NGS workflow consisted of library preparation step, template preparation step, and sequencing in Ion Proton semiconductor sequencer. Sequences were then analyzed using the SNP & Variation Suite (Golden Helix, Inc.). The target genes were as follow: *ACTG1*, *CCDC50*, *CDH23*, *CEACAM16*, *CLDN14*, *COCH*, *COL11A2,* *CRYM*, *DFNA5*, *DFNB59*, *DIABLO (SMAC)*, *DIAPH1*, *ESPN*, *ESRRB*, *EYA4*, *GIPC3*, *GJB2*, *GJB3*, *GJB6*, *GPSM2*, *GRHL2*, *GRXCR1*, *HGF*, *ILDR1*, *KCNQ4*, *LHFPL5*, *LOXHD1*, *LRTOMT*, *MARVELD2*, *MIR96*, *MITF*, *MSRB3*, *MYH14*, *MYH9*, *MYO15A*, *MYO1A*, *MYO3A*, *MYO6*, *MYO7A*, *OTOA*, *OTOF*, *PCDH15*, *POU3F4*, *POU4F3*, *PRPS1*, *PTPRQ*, *RDX*, *SERPINB6*, *SLC17A8*, *SLC26A4*, *SLC26A5*, *SMPX*, *STRC*, *TECTA*, *TJP2*, *TMC1*, *TMIE*, *TMPRSS3*, *TPRN*, *TRIOBP*, *USH1C*, *USH2A*, *WFS1*, and *WHRN*.

### Plasmids, cell culture, transfection, and reporter assay

The luciferase reporter containing the human *TYR* promotor (pGL3-Tyr-Luc; − 1995/+ 74) was kindly donated by Dr. Reinisalo, University of Eastern Finland, Finland (Fig. 3c)^22^. A wild-type *MITF* expression plasmid was prepared from a full length *MITF*-isoform A cDNA, which was obtained from pCMV3-C-GFPSpark-MITF (Sino Biological Inc., China) followed by HindIII and XbaI restriction digestion and ligation into pCMV10 3X Flag (N-terminal; Sigma, St Louis, WA, USA). Site-directed mutagenesis was performed using QuikChange II XL Site-Directed Mutagenesis Kit (Agilent, USA), according to the manufacturer’s protocol. All plasmid constructs were selected with diagnostic restriction digestion and verified by Sanger sequencing. Wild-type and mutant constructs were cloned into plasmid pCMV10 3X Flag and pCMV B-galactosidase (kindly provided by Dr. Jia-da Li, Central South University of China, China) before transient transfection for luciferase and beta-galactosidase activity assays, as previously described^15^.

A 1 × 10^5^ melanoma (G361) or mouse embryonic fibroblast (NIH/3T3) cells were seeded into 6-well plates and cultivated in Dulbecco’s modified Eagle’s medium supplemented with 10% fetal bovine serum, 100 U/ml penicillin and 100 μg/ml streptomycin, under 5% CO_2_ at 37 °C for 24–48 h prior to transfection. Cells were 70–80% confluent on the day of transfection. The transfection mix was prepared using a ratio of 3 μl Lipofectamine 2000 for every 1 μg of total DNA transfected. pCMV10 3X Flag and pCMV B-galactosidase were used for normalization and monitoring the transfection efficiency, respectively.

Twenty-four hours after transfection, lysates were prepared from the cultured G361 cells (Promega, USA) and then assayed for luciferase and B galactosidase activity using the Infinite 200 PRO plate reader (Tecan, Switzerland). All reporter assays were performed in triplicate, at least three times on different days, using different batches of cells. The G361 cells were kindly provided by Prof. Uraiwan Panich (Mahidol University) and the NIH3T3 cells were purchased from ATCC.

### Western blot analysis

The MITF protein in transfected G361 cells was verified by western blot analysis using a mouse monoclonal anti-Flag M2 antibody. Cells cultured for 48 h were harvested and protein isolated using RIPA lysis buffer (Bio-Rad), supplemented with protease inhibitor cocktail (AppliChem, Germany) and 1 mM phenylmethylsulfonyl fluoride (Merck, Germany). The proteins (20 µg) were then subjected to SDS-PAGE (12%) and transferred onto a polyvinylidene fluoride membrane with pore size 0.22 µm. The membrane was blocked using blocking solution (3% bovine serum albumin in TBS plus 0.1% Tween-20) for 2 h and then stained with a mouse monoclonal anti-Flag M2 antibody (1:1,000 dilution, Sigma) at 4 °C overnight. A goat anti-mouse GAPDH antibody (Thermo Scientific) was used as a control for protein loading. After washing with washing buffer (TBS plus 0.1% Tween-20) for 15 min, four times, the membrane was stained for 1 h with a goat anti-mouse IgG secondary antibody conjugated with horseradish peroxidase (1:1,000 dilution, Thermo Fisher Scientific) at room temperature followed by four washes. Protein levels were measured using Luminata Forte Western HRP substrate (Merck, USA) with a C-DiGit Chemiluminescence Western Blot Scanner (LI-COR Bioscience).

### Immunofluorescence study

NIH3T3 cells were transfected with 200 ng of pCMV10 3X Flag wild-type MITF or MITF variant expression plasmids following an established protocol^15^. At 48 h after transfection, the cells were fixed with 4% paraformaldehyde at room temperature for 30 min, permeabilized in PBS plus 0.2% Triton X-100 (Scharlau, Spain) for 1 h, blocked with blocking solution (PBS, 3% bovine serum albumin plus 5% goat serum) at room temperature for 1 h, stained with mouse monoclonal anti-Flag M2 primary antibody (1:600 dilution, Sigma) at 4 °C overnight, washed with PBS for three times, and then incubated for 2 h with DyLight 488 fluorescence-labeled secondary goat anti-mouse antibody (1:300 dilution, Thermo Fisher Scientific). The cells were incubated with 4,6-diamino-2-phenylindole (DAPI, Invitrogen) for 3 min before immunofluorescence analysis using a laser scanning confocal microscope (Nikon, Japan) and the NIS-Elements Viewer software package.

### Molecular modeling

Structural models of human wild-type MITF and Arg341His and Arg341Cys MITF variants were generated with SWISS-MODEL^36,37^ using the *Mus musculus* MITF:E-box complex (4atk) and apo MITF (4ath) as templates^10^. Structures were viewed using the PyMOL Molecular Graphics System, version 2.0.6 (Schrödinger, LLC, NY, USA).

## Supplementary information

Supplementary information.

## Data Availability

Data openly available in a public repository that issues datasets with DOIs.
