# Supplementary material for: MITF variants cause nonsyndromic sensorineural hearing loss with autosomal recessive inheritance
Source: Sci Rep. 2020 Jul 29;10:12712. doi: 10.1038/s41598-020-69633-4 (PMC7391749; doi:10.1038/s41598-020-69633-4)
Supplement: Supplementary file 1 — Supplementary information. [file 41598_2020_69633_MOESM1_ESM.pdf]

Supplementary Information

MITF variants cause nonsyndromic sensorineural hearing loss with autosomal recessive inheritance

Supranee Thongpradit[1,2], Natini Jinawath[3,4], Asif Javed[5,6], Saisuda Noojarern[7], Arthaporn Khongkraparn[7], Thipwimol Tim-aroon[7], Krisna Lertsukprasert [8], Bhoom Suktitipat[4,9], Laran T Jensen[10], Duangrurdee Wattanasirichaigoon[7*]

[1] Program in Molecular Medicine, Faculty of Science, Mahidol University, Bangkok, Thailand

[2] Research Center, Faculty of Medicine Ramathibodi Hospital, Mahidol University, Bangkok, Thailand

[3] Program in Translational Medicine, Faculty of Medicine Ramathibodi Hospital, Mahidol University, Bangkok, Thailand

[4] Integrative Computational Bioscience Center, Mahidol University, Salaya, Nakhon Pathom, Thailand

[5] Computational and Systems Biology Group, Genome Institute of Singapore, Agency for Science, Technology and Research, Singapore

[6] School of Biomedical Sciences, University of Hong Kong, Hong Kong

[7] Division of Medical Genetics, Department of Pediatrics, Faculty of Medicine Ramathibodi Hospital, Mahidol University, Bangkok, Thailand

[8] Department of Communication Sciences and Disorders, Faculty of Medicine Ramathibodi Hospital, Mahidol University, Bangkok, Thailand

[9] Department of Biochemistry, Faculty of Medicine Siriraj Hospital, Mahidol University, Bangkok, Thailand

[10] Department of Biochemistry, Faculty of Science, Mahidol University, Bangkok, Thailand

*Corresponding author

Supplementary Methods

Otogenome hearing loss gene panel analysis

DNA was isolated from leukocytes using the Purgene® DNA extraction kit .The integrity and purity of DNA was confirmed by gel electrophoresis. Concentrations were determined on a Qubit fluorometer using a dsDNA BR kit (Invitrogen, Q32850).

HL gene panel, OtoGenome^{TM}, (Laboratory for Molecular Medicine, Cambridge, MA, USA) consisted of 70 HL genes: *ACTG1*, *ATP6V1B1*, *BSND*, *CCDC50*, *CDH23*, *CLDN14*, *CLRN1*, *COCH*, *COL11A2*, CRYM, *DFNA5*, *DFNB31*, *DFNB59*, *DIAPH1*, *ESPN*, *ESRRB*, *EYA1*, *EYA4*, *GIPC3*, *GJB2*, *GJB3*, *GJB6*, *GPR98*, *GPSM2*, *GRHL2*, *GRXCR1*, *HGF*, *ILDR1*, *KCNE1*, *KCNQ1*, *KCNQ4*, *LHFPL5*, *LOXHD1*, *LRTOMT*, *MARVELD2*, *MIR96*, *MIR183*, *MSRB3*, *MTRNR1* (12S rRNA), *MTTS1* [tRNAser (UCN)], *MYH14*, *MYH9*, *MYO15A*, *MYO1A*, *MYO3A*, *MYO6*, *MYO7A*, *OTOA*, *OTOF*, *POU3F4*, *POU4F3*, *PRPS1*, *RDX*, *SERPINB6*, *SLC17A8*, *SLC26A4* (*PDS*), *STRC*, *TECTA*, *TIMM8A*, *TJP*, *TMC1*, *TMIE*, *TMPRSS3*, *TPRN*, *TRIOBP*, *USH1C*, *USH1G*, *USH2A*, and *WFS1*. DNA capture and amplification from genomic DNA was performed using oligonucleotide base target capture (Agilent SureSelect) followed by Illumina HiSeq sequencing of the coding regions and splice sites of the genes. Sanger sequencing was used to confirm all clinically significant variants and to fill in regions with insufficient coverage. Variants classified as likely benign or benign were not confirmed. Variant calls were generated using the Burrows-Wheeler Aligner followed by GATK analysis.

144 nonsyndromic hearing loss genes analyzed (https://www.fulgentgenetics.com/Hearing-Loss-Nonsyndromic, https://hereditaryhearingloss.org)

ACTG1, ACTG10, ACTG11, ACTG12, ACTG13, ACTG14, ACTG15, ACTG16, ACTG17, ACTG18, ACTG19, ACTG2, ACTG20, ACTG21, ACTG22, ACTG23, ACTG24, ACTG25, ACTG3, ACTG4, ACTG5, ACTG6, ACTG7, ACTG8, ACTG9, ADCY1, AIFM1, BDP1, BSND, CABP2, CCDC50, CD164, CDC14A, CDH23, CEACAM16, CIB2, CLDN14, CLIC5, COCH, COL11A1, COL11A2, COL4A6, CRYM, DCDC2, DFNA5, DFNB59, DIAPH1, DMXL2, ELMOD3, EPS8, EPS8L2, ESPN, ESRP1, ESRRB, EYA4, FAM65B, FOXI1, GIPC3, GJB2, GJB3, GJB6, GPSM2, GRHL2, GRXCR1, GRXCR2, GSDME, HARS2, HGF, HOMER2, IFNLR1, ILDR1, KARS, KCNQ4, KITLG, LHFPL5, LMX1A, LOXHD1, LRTOMT, MARVELD2, MCM2, MET, MIR96, MIRN96, MITF, MPZL2, MSRB3, MYH14, MYH9, MYO15A, MYO1A, MYO3A, MYO6, MYO7A, NARS2, NLRP3, OSBPL2, OTOA, OTOF, OTOG, OTOGL, P2RX2, PCDH15, PDE1C, PDZD7, PJVK, PNPT1, POU3F4, POU4F3, PPIP5K2, PRPS1, PTPRQ, RDX, REST, RIPOR2, ROR1, S1PR2, SERPINB6, SIX1, SLC17A8, SLC22A4, SLC26A4, SLC26A5, SLITRK6, SMAC, SMPX, STRC, SYNE4, TBC1D24, TECTA, TJP2, TMC1, TMC2, TMEM132E, TMIE, TMPRSS3, TNC, TPRN, TRIOBP, TSPEAR, USH1C, USH2A, WBP2, WFS1, WHRN

Table S1 Variants of hearing loss genes identified in exome sequences of the affected and unaffected individuals from Family-1

Gene	RefSeq transcript ID	Inheritance	Coding	Protein	Affected			Unaffected	Allele feq		SNPs ID	ACMG[b]	Classification
					IV-2	IV-4	IV-5	IV-7	In-house[a]	gnomAD			
ELMOD3	NM_001135023	AR	c.313A>G	p.Ser105Gly	Het	WT	Het	Het	0.0658	0.011	rs78809694	BA1, BP1, BP4, BP6	Benign
ESRRB	NM_004452	AR	c.16A>G	p.Arg6Gly	WT	WT	WT	Het	0.0416	0.00317	rs143477571	PP3, BS1, BS2, BP1, BP6	Benign
GJB2	NM_004004	AD/AR	c.109G>A	p.Val37Ile	Het	WT	Het	WT	0.0827	0.00772	rs72474224	PS1, PS3, PM1	Pathogenic
GRHL2	NM_024915	AD	c.1243G>A	p.Val415Ile	Het	WT	Het	WT	0.0434	0.0135	rs3779617	PP3, BA1, BP1, BP6	Benign
MCM2	NM_004526	AD	c.1999G>A	p.Val667Met	Het	WT	Het	WT	0.0439	0.00338	rs2307311	PP3, BS1, BS2, BP1, BP6	Benign
MITF	NM_198159.3	AD	c.1022G>A	p.Arg341His	Hom	Hom	Hom	WT	0	0.00000399	rs1195515853	PM1, PM2, PM5, PP3	Likely-pathogenic
MYH9	NM_002473	AD	c.2635A>C	p.Met879Leu	Het	Het	Het	WT	0.00288	0.000231	rs200328859	PP2, BS1, BS2, BP4, BP6	Benign
MYO7A	NM_001127179	AD/AR	c..4805G>A	p.Arg1602Gln	Het	WT	Het	Het	0.0271	0.00371	rs139889944	PM1, BS1, BS2	Benign
OTOA	NM_144672	AR	c.2359G>T	p.Glu787*	WT	WT	Het	WT	0.182	0.000308	rs200988634	PVS1, PP3	Uncertain Significance
OTOGL	NM_173591	AR	c.139C>T	p.Gln47*	WT	WT	Het	Het	0	0.0000101	rs1193594940	PVS1, PM2, PP3	Pathogenic
PCDH15	NM_033056	AR	c.2884C>T	p.Arg962Cys	WT	Het	Het	WT	0.0271	0.000921	rs201816080	PM1, PM2, PP3, BP1	Uncertain Significance
REST	NM_001193508	AD	c.2241A>G	p.Ile747Met	WT	Het	Het	WT	0	0	rs144031960	PM2, BP1, BP4	Likely-benign
TECTA	NM_005422	AD/AR	c.5372C>G	p.Pro1791Arg	Het	WT	WT	WT	0.00107	0.0000868	rs754213928	PM1, PM2, PP2, PP5	Likely-pathogenic
TMC2	NM_080751	AD	c.2548_2550delAAG	p.lys850del	Het	Het	Het	Het	0.0507	0.0109	rs142900000	PM4, PP3, BA1	Benign
TRIOBP	NM_001039141	AR	c.5764C>T	p.Arg1922Trp	WT	Het	WT	Het	0.0137	0.00118	rs183941928	PM2, PP3, BP1, BP6	Likely-benign
			c.163G>T	p.Val55Leu	WT	Het	WT	WT	0.07	0.0147	rs77782321	PM1, BA1, BP4, BP6	Benign

[a] in 200 individuals who did not have hearing loss as a major manifestation

[b] Richards, S. *et al.* Standards and guidelines for the interpretation of sequence variants: a joint consensus recommendation of the American College of Medical Genetics and Genomics and the Association for Molecular Pathology. *Genet Med.* **17,** 405-424 (2015).

Table S2 Top 17 genes ranked by PhenGen analysis showing scores, gene location and corresponding disorders/major protein functions.

Gene	Probability damaging score	Location	Corresponding disorders /protein functions
MITF	0.0885690996	3p14.1	WS2A, COMMAD, ADFNS
KCNJ12	0.0055963440	17p11.2	Andersen cardiodysrhythmic periodic paralysis
MUC6	0.0025231421	11p15.5	major function in secreted mucins of the digestive tract
NAV3	0.0011328982	12q14.3-q21.1	involved in the process of neuron growth and regeneration
NDST3	0.0010090281	4q26	catalyzing N-deacylation and sulfate transfer in N-acetyl glucosamine subunits of heparin sulfate
AP1S3	0.0009123732	2q36.1	pustular psoriasis
KIAA0430	0.0008699896	16p13.13	involved in ncRNA splicing
DNHD1	0.0007344898	11p15.4	unknown
RAD21-AS1	0.0006839718	-	ncRNA
uc003dws.3	0.0006839718	-	ncRNA
*OLFM*1	0.0005252689	9q34.3	regulating the production of neural crest by the neural tube
NM_001100111	0.0002433756	-	ncRNA
BAGE	0.0001904779	21p11.1	unknown
MUC20	0.0000086459	3q29	regulate the Met signaling cascade through suppression of the Grb2-Ras pathway
ZNF717	0.0000042796	3p12.3	may function as a transcriptional factor
NISCH	0.0000023135	3p21.1	regulate cell migration by inhibiting p21-activated kinase (PAK1)
MADCAM1	0.0000022778	19p13.3	membrane bound receptor regulating of leukocytes in mucosal tissues

Table S3 Primer sequences and PCR conditions for *MITF*-isoform A[a]

Exon	Sequence (5′-3′)	Annealing temperature (°c)
MITF-M 1F [b]	CAAACTCGTAGGGCTTCCAA	60
MITF-M 1R [b]	TGAGCAATGAACAGGAGCTG	
1F	TGGGAGCTGTAGTTTTCGTG	63
1R	CTCTCCTCGCCCCAGAGT	
2F	TGGAGTACCATTCTGTGACTTGA	60
2R	ACACCTAGCAAATGGAAAATGG	
3F	TCATGTTTGTGCCTGAAGGA	60
3R	CTTTTCCACCTCCCCTTCTC	
4F	CTGTGCCATCAGCTTTGTGT	60
4R	TGCTTAAGTTTTCAGGAAGGTG	
5F	CCAATGCTTGGCAACTCATA	60
5R	GAACCCTGGAAACACCTCAA	
6F	CAAAGGGAACTGGTTGAGGA	60
6R	TGTTTTAACCACTGCAGAGACC	
7F	GCTTTTGAAAACATGCAAGC	60
7R	CAGCTGTAGGAATCAACTCTCC	
8F	CGTTGTCATGACCTGGAGAA	60
8R	CCTTTTGCACAATTCAATGC	
9F	GTACACGGCTTGGGTGGT	60
9R	GTCAACTCCCCTATGGCTCA	
10pF	CTAATGACGCGCATCTACCA	60
10pR	TCCTGGGCTATTGATAAAGCA	
10dF	AACTGCTTCCTTTCTTGATTCG	60
10dR	CCCTTCAGTTTCGGTTGGTA	

[a] PCR condition for MITF-isoform A and exon 1 of MITF-isoform M: initial denaturation at 94°C 5 min; 35 cycles of denaturation at 94°C for 45 sec, annealing for 45 sec, extension at 72°C for 45 sec (but 2 min for exon 1 of MITF-isoform A); and final extension at 72°C for 7 min. [b] primers sequence for exon 1 of *MITF*-M isoform. Reference sequences for MITF-A: NT_011520.11, NM_006941.3, NP_008872.1; for isoform M: *MITF* isoform-M: NT_022495, NM_000248, NP_000239

Table S4 Primer sequences and PCR conditions for *GJB2*

Exon	Sequence (5′-3′)	Annealing temperature (°c)
1F	TCTTTTCCAGAGCAAACCGCC	60
1R	R1-TGAGCACGGGTTGCCTCATC	

Table S5 Primer sequences and PCR conditions for *TYR* mutation screening[a]

Exon	primer name	primer sequence (5'-3')	Annealing temperature (°C)
1	1-1F	TGTGCTTTTCAGAGGATGAA	50
	1-1R	CAAACTTGCAGTTTCCACAG	
	1-2F	TCTGTCCAATGCACCACTT	50
	1-2R	GCTTCTGGATTTCTTGTTCC	
	1-3F	CCCATGTTTAACGACATCAA	50
	1-3R	ATACCCTGCCTGAAGAAGTG	
2	2F	CTCAGGAGAAGTCTAACAAC	50
	2R	AACTCAGAAAATTCTGAATTC	
3	3F	GCCATTTAATTCCACAAAT	50
	3R	GGTGACAACCTGATCACAGA	
4	4F2	ATTTTGCAGCATTCTGGAGG	55
	4R2	TGCCATGTACAAAATGGCCT	
5	5F	GAGGCTTTGGAGTATTAGGTG	50
	5R	GTTCCTCATTACCAAATAGCA	

[a] PCR 35 cycles; reference sequences of TYR Variant 1: NT_008984, NM_000372, NP_000363; gDNA 118.21 Kb; mRNA 2384 bp; CDS 1590 bp

Figure S1 IGV data revealing the depth coverage varying between 92 and 152 reads, suggesting no missed call of the *MITF* segment.

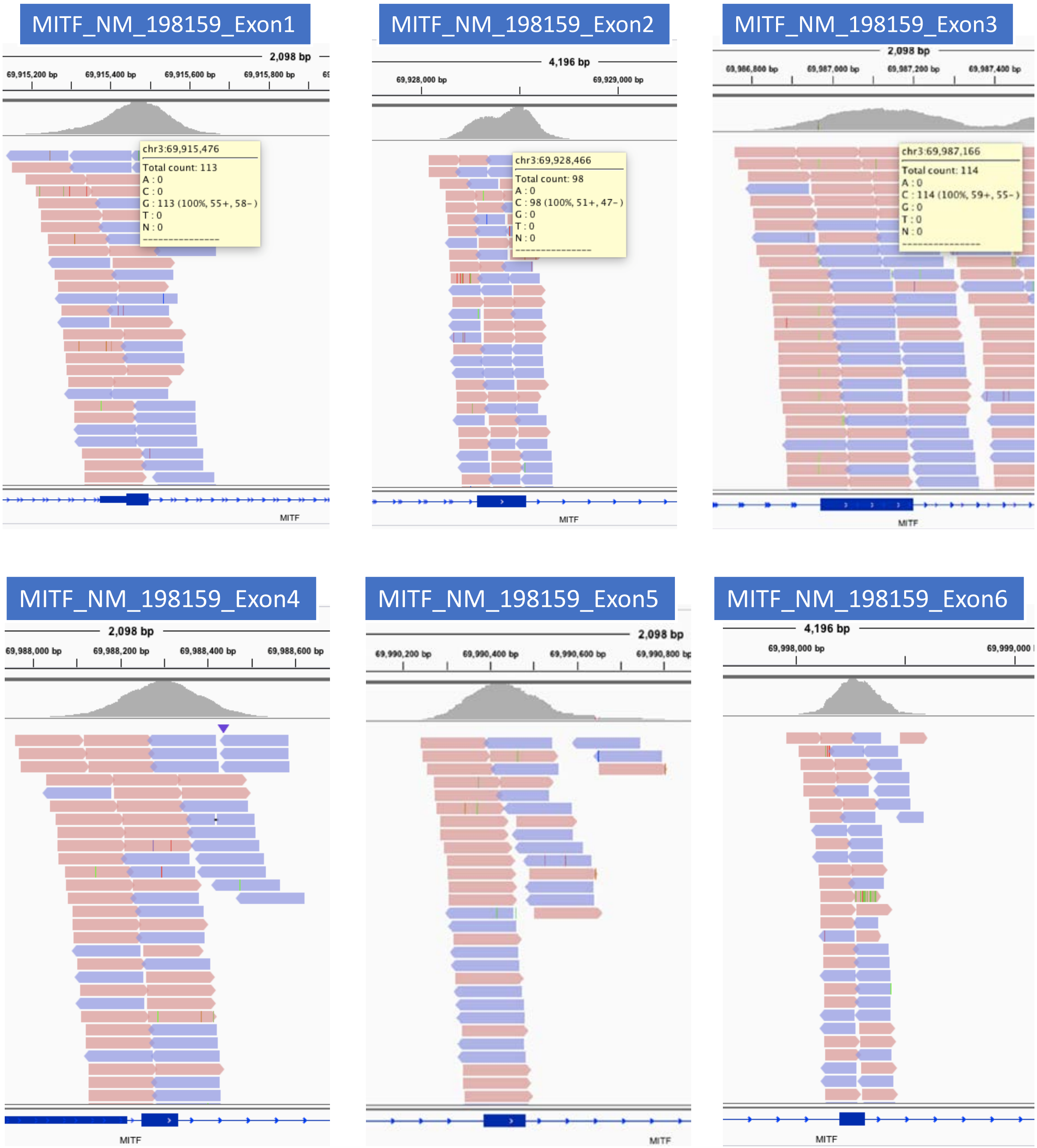

MITF_NM_198159_Exon7

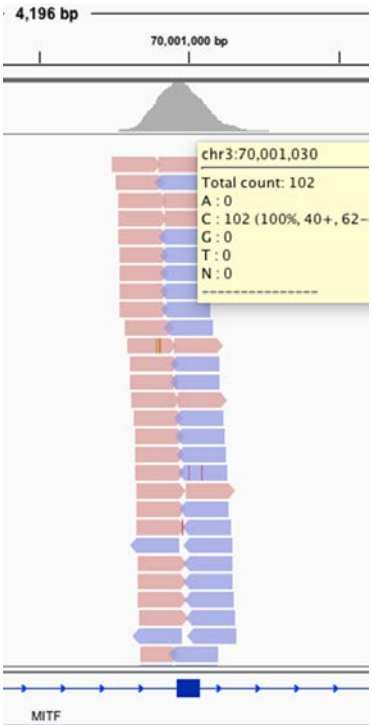

MITF_NM_198159_Exon8

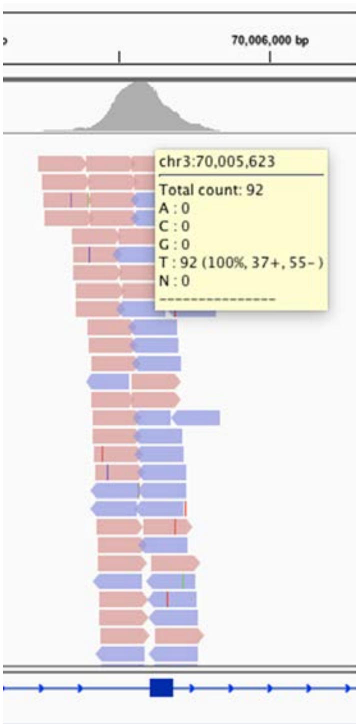

MITF_NM_198159_Exon9

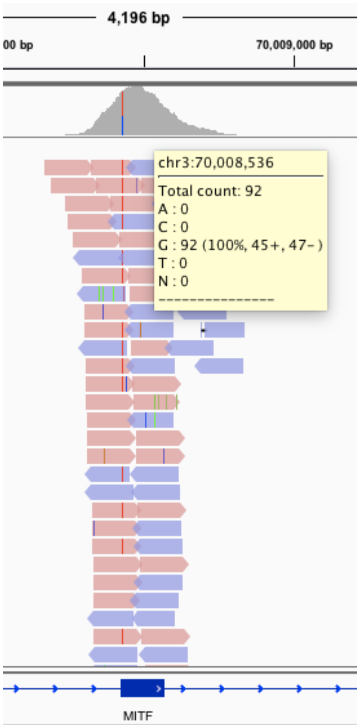

MITF_NM_198159_Exon10

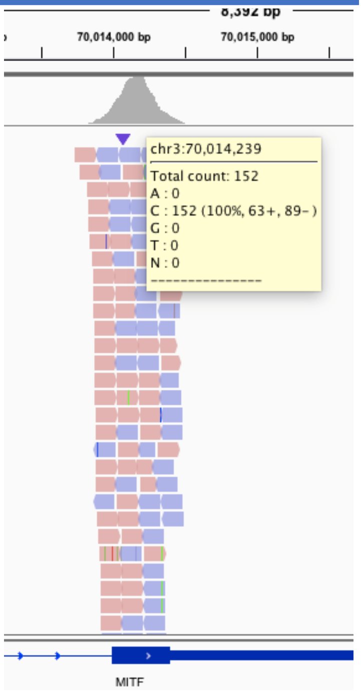

Figure S2 Western blot of MITF wildtype and R341C and R341H. (a) A full-length gel showing, from left to right, molecular size marker; wildtype MITF-isoform A, R341C, R341H-1, and R341H-2 at around 105 kD; and wildtype MITF-isoform M (smaller size compared to MITF-isoform A). There was a significant increase of the R341C protein expressed, and a slightly decreased quantity of the R341H variant compared with wild-type MITF-A. The specimens of WT-A isoform, R341C, R341H-2 and MITF-M isoform were from transfection experiments performed on the same day, whereas that of R341H-1 was from an earlier experiment (one month apart). Our reason of having R341H-1 and R341H-2 on side-by-side was to confirm the decreased protein expression detected in our study. The 40^{+} and 30^{+} kD bands represented represented nonspecific background. (b) Lower part of the full-length gel [(of fig (a)] showing amount GAPDH used as a loading control for normalization for wildtype MITF and each variant. Noted almost equivalent amount of the GAPDH in all, but MITF-M, lanes. Details of preparation of this blot were as follow: the lower part of the full-length gel was cut out, followed by washing with PBST, three times; then incubated with GAPDH, washed with PBST before staining with secondary antibody; and washed again with PBST prior to visualization. Brightness and contrast of the images were not modified from its original appearances. Size marker used was PM2510, ExcelBand™, SMOBIO.

(a)

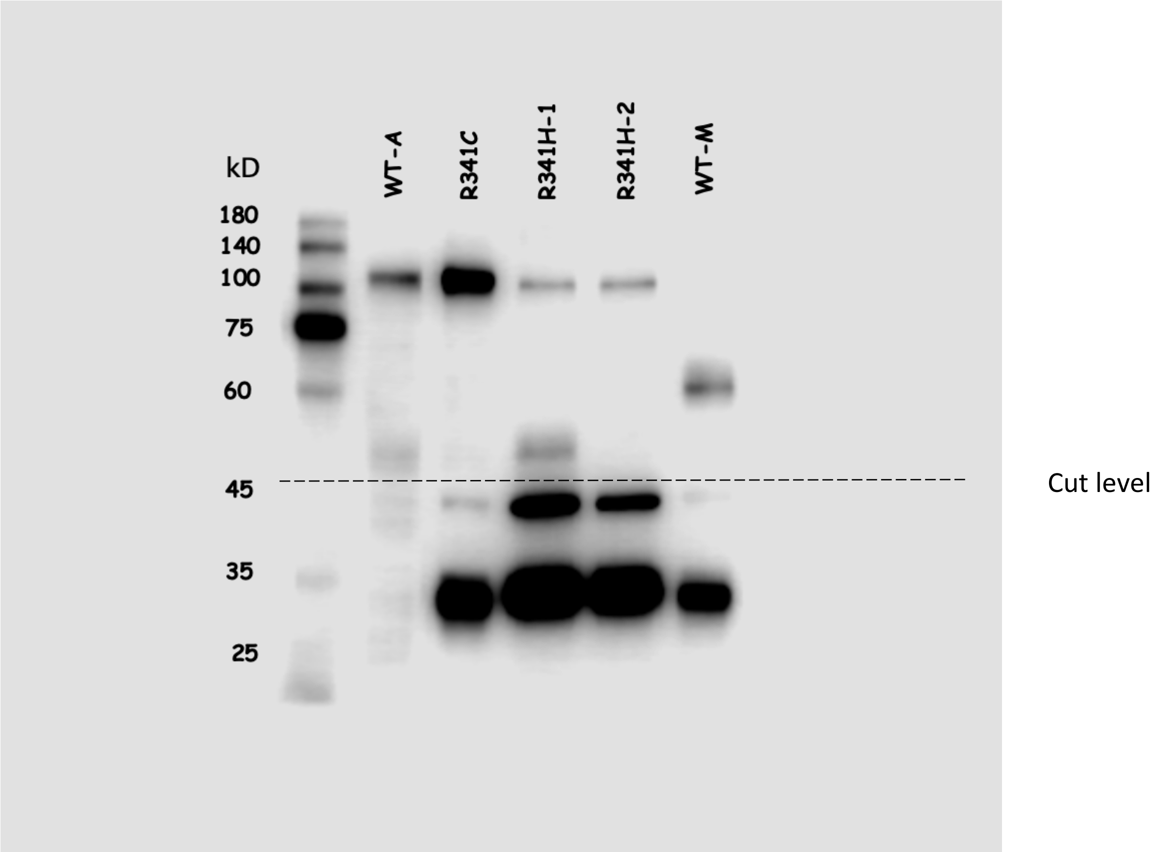
kD
180
140
100
75
60
45
35
25
WT-A
R341C
R341H-1
R341H-2
WT-M
Cut level

(b)

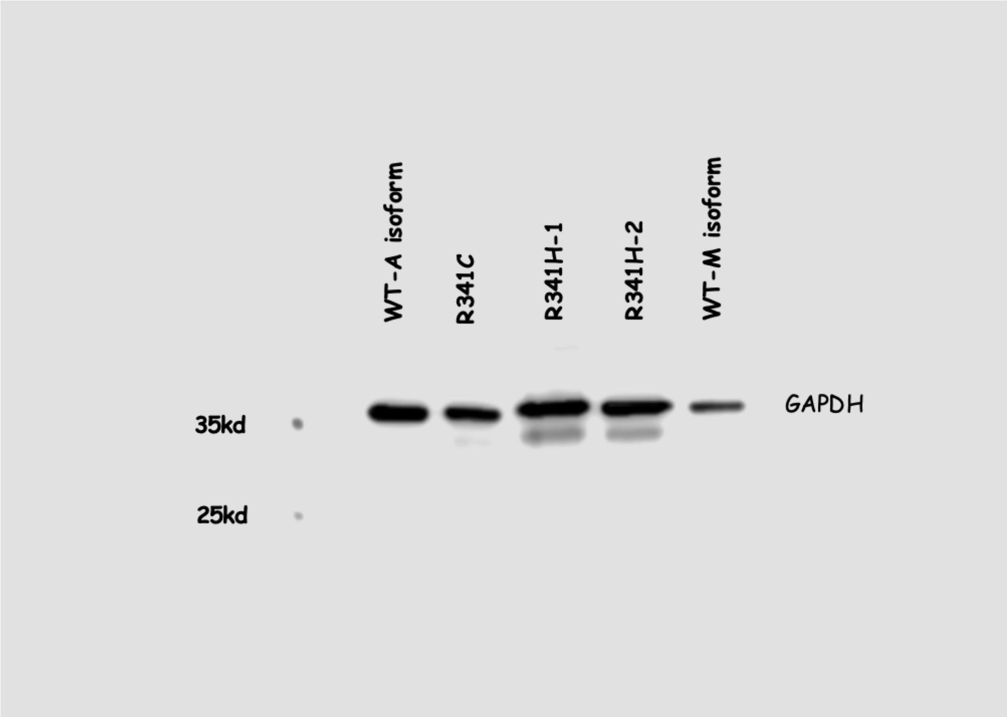
WT-A isoform
R341C
R341H-1
R341H-2
WT-M isoform
35kd
25kd
GAPDH
